# Patient-Derived Xenograft Models in Cancer Research

**DOI:** 10.3390/cancers13040815

**Published:** 2021-02-16

**Authors:** Wytske M. van Weerden

**Affiliations:** Department of Urology, Erasmus MC Cancer Institute, Room BE362, Erasmus University Medical Center, P.O. Box 2040, 3000 CA Rotterdam, The Netherlands; w.vanweerden@erasmusmc.nl; Tel.: +31-10-704-3674

**Keywords:** patient-derived xenograft (PDX), tumor biology, tumor microenvironment, metastasis, tumor immunology, thin-cut tissue slices, organoids

This series of 12 articles, consisting of 9 original articles and 3 reviews, is presented by international leaders in translational cancer research. This series highlights the various efforts to establish valuable panels of patient-derived xenograft (PDX) models from various cancer types and relevant tools to apply translational research. We are especially pleased to also have reports in this series which focus on important new developments to serve as alternative patient-specific model systems. 

Patient-directed (personalized) medicine is an attractive concept that is embraced by clinicians to improve therapeutic success. PDXs are considered to retain the unique genetic characteristics of the patient tumor, therewith enabling the prediction of patient-specific tumor responses. This Special Issue includes three reviews describing the potential translational power of current PDXs for head and neck squamous cell carcinoma [1], pancreatic cancer [2], urothelial cell cancer and renal cell cancer [3]. These reports highlight the pros and cons of PDX models in general and more specifically of their utility in the respective research areas, to discover novel targets to aid precision medicine and to develop prediction biomarkers to stratify patients for individualized therapy. 

The original papers by Meneceur et al. [4] and Oshi et al. [5] describe the development of a panel of PDXs directly from patients with glioblastoma and breast cancer brain metastasis, respectively, and focus on the characterization of these models and their ability to represent the patient’s original tumor features. Interestingly, Oshi et al. [5] point to the highly relevant issue of whether PDX models often derived from primary tumors and propagated subcutaneously faithfully reflect the metastatic disease—in this case, brain tumors. They effectively show the feasibility to establish such primary PDX orthotopic models in the mouse brain and provide evidence that therapy response was affected by the site of implantation being orthotopic or ectopic in the mouse mammary fat pad. These data underscore the relevance of the metastatic microenvironment on therapy responses, as well as the need to use high fidelity site-specific metastatic PDX models to adequately reflect this stage of disease.

The importance of the tumor niche on drug response was also investigated in PDX models of acute myeloid leukemia described in the contribution by Schueler et al. [6]. They describe the establishment and characterization of a panel of 20 PDXs and identified that tumor growth kinetics of various PDXs were dependent on the injection site of the tumor cells when inoculated into bone or spleen or subcutaneously. Subsequently, and confirming the earlier findings by Oshi et al. [5], sensitivity to relevant chemotherapeutics was shown to be different in the various sites, underscoring the impact of the tumor microenvironment on drug sensitivity. These reports provide a relevant warning that the interpretation of outcome data requires comprehensive knowledge of the specific characteristics of the PDX model used and its translational limitation.

Considerations regarding the fitness of traditional PDXs as tools for response prediction especially also relate to their use as platforms to evaluate the efficacy of immunotherapies. Liu et al. [7] describe the establishment of the first PDX model of nasopharyngeal carcinoma in human immune cell-engrafted immunocompromised mice. This humanized PDX model revealed prominent tumor-infiltrating T cells reflective of clinical cancer, which, after standard combination immune therapy with checkpoint inhibitors, were re-activated, suggestive of restored T-cell function. Although these chimeric human immune models have been received with great enthusiasm, Liu et al. rightfully point to the current limitations regarding the absence of full HLA matching and incomplete education and maturation of human pre-T cells in the mouse thymus.

This series also includes four original papers describing the use of alternative PDX-based systems for therapy response prediction. One interesting application of PDX xenografted tumors is the use of thin-cut tissue slice cultures (TSC). TSC has the advantage of relatively fast and standardized culture procedures with maintenance of the original 3D architecture and essential cell interactions. Suckert et al. [8] describe in their contribution the use of TSC of PDXs to evaluate proton beam radiotherapy responses of squamous cell carcinoma of the head and neck. While reporting proton-irradiated TSC of PDX tumors to resemble tumor morphology changes and DNA damage comparable to that observed in vivo, they also discuss relevant experimental aspects, such as the need for consecutive slices to capture tumor tissue heterogeneity and sufficient replicate numbers to reflect variability of response. 

Another interesting alternative PDX platform to mouse-based PDX models that has been pursued intensely for various cancer types over the last 15 years is the zebrafish (ZF) xenotransplantation model. Like PDXs in immune-compromised mice, patient-derived xenografted tumor tissues in zebrafish embryos have been suggested as attractive models (so-called avatars) to facilitate co-clinical trials and implement personalized medicine. In this special series, we have two papers that address highly relevant issues related to the utility and translational power of these PDX systems. The paper by Usai et al. [9] offers an important contribution to the ZF avatar application by defining a general criterion for chemotherapeutic dose conversions from humans to fish in order to allow transferability. Data obtained from safety testing of 10 different chemotherapy regimens used in cancer treatment in zebrafish embryos were matched with efficacy testing using cell line-derived xenograft (CDX) models, generating a basic formula for dose equivalence that was validated in newly established zebrafish PDXs of fresh surgical specimens of patients with colon, pancreatic, or gastric cancers. Gauert et al. [10] concentrated on the challenge of establishing a PDX platform with a sufficient time window to allow co-clinical trials to serve as prediction models in personalized medicine. Here, they describe the successful application of ZF-based PDXs of acute lymphoblastic leukemia to predict patient-specific drug responses in a clinically relevant time window of 5 days. The report details drug responses at five days’ treatment of fresh ZF-PDXs from newly diagnosed and relapse patients, reflecting those observed in the clinic, providing proof-of-concept for their standardized assay based on quantifiable read-outs to be used as prediction tools. 

Finally, another alternative PDX platform is provided in the contribution by Winter et al. [11] using the Hen’s egg test–chorioallantoic membrane (HET–CAM) PDX model. Like the ZF embryo, the chicken embryo lacks a functional immune system and is considered a non-life animal, making these PDX models an attractive platform for (“non-animal”) drug testing. While HET–CAM has been predominantly used to investigate vasculature and angiogenesis-targeted interventions, Winter et al. describe the feasibility of high-resolution MRI and PET-imaging methods to evaluate novel radiotracers in this CDX-based HET–CAM model. 

To finish, this collection on PDX models is completed by the report by O’Farrell et al. [12], who offer a systems modelling approach to assess chemotherapy sensitivity in PDXs and apply these data to identify response biomarkers and stratify patients for therapy. They describe the use of protein profiling combined with computational modelling to identify drug responsive PDX tumors and used tumor growth, 18F-Fluorodeoxyglucose positron emission tomography/computed tomography (18F-FDG-PET/CT), and CT analysis (radiomics) to validate drug sensitivity prediction. This study emphasizes the value of various endpoint parameters that independently may predict tumor responses that could not have been distinguished based on each feature alone. This systems modelling approach highlights the value of variable tumor parameters, including high-content (imaging) data, to predict drug sensitivity. 

This series of articles demonstrates the wide use of PDX models in a broad and diverse field of research. It contains detailed descriptions for the development of PDXs for a specific cancer type, while others report on more generally applicable issues regarding the utility of PDX models. Besides the use of the immune-compromised mouse as a host for PDXs, several papers highlight alternative systems for patient-specific tissue engraftment. Collectively, these articles are a valuable asset to the field of PDX research, with the overall goal to aid patient-directed (personalized) medicine and to improve therapeutic success.
